# Plasmalogens Eliminate Aging-Associated Synaptic Defects and Microglia-Mediated Neuroinflammation in Mice

**DOI:** 10.3389/fmolb.2022.815320

**Published:** 2022-02-23

**Authors:** Jinxin Gu, Lixue Chen, Ran Sun, Jie-Li Wang, Juntao Wang, Yingjun Lin, Shuwen Lei, Yang Zhang, Dan Lv, Faqin Jiang, Yuru Deng, James P. Collman, Lei Fu

**Affiliations:** ^1^ School of Pharmacy, Shanghai Jiao Tong University, Shanghai, China; ^2^ Wenzhou Institute, University of Chinese Academy of Sciences, Wenzhou, China; ^3^ Department of Chemistry, Stanford University, Stanford, CA, United States; ^4^ Academy of Pharmacy, Xi’an Jiaotong-Liverpool University, Suzhou, China

**Keywords:** aging, plasmalogen, synaptogenesis, neurogenesis, microglia, neuroinflammation

## Abstract

Neurodegeneration is a pathological condition in which nervous system or neuron losses its structure, function, or both leading to progressive neural degeneration. Growing evidence strongly suggests that reduction of plasmalogens (Pls), one of the key brain lipids, might be associated with multiple neurodegenerative diseases, including Alzheimer’s disease (AD). Plasmalogens are abundant members of ether-phospholipids. Approximately 1 in 5 phospholipids are plasmalogens in human tissue where they are particularly enriched in brain, heart and immune cells. In this study, we employed a scheme of 2-months Pls intragastric administration to aged female C57BL/6J mice, starting at the age of 16 months old. Noticeably, the aged Pls-fed mice exhibited a better cognitive performance, thicker and glossier body hair in appearance than that of aged control mice. The transmission electron microscopic (TEM) data showed that 2-months Pls supplementations surprisingly alleviate age-associated hippocampal synaptic loss and also promote synaptogenesis and synaptic vesicles formation in aged murine brain. Further RNA-sequencing, immunoblotting and immunofluorescence analyses confirmed that plasmalogens remarkably enhanced both the synaptic plasticity and neurogenesis in aged murine hippocampus. In addition, we have demonstrated that Pls treatment inhibited the age-related microglia activation and attenuated the neuroinflammation in the murine brain. These findings suggest for the first time that Pls administration might be a potential intervention strategy for halting neurodegeneration and promoting neuroregeneration.

## 1 Introduction

Plasmalogens (Pls) are a special type of vinyl ether-bonded phospholipids actively participating in structure and function of biological membranes. Approximately 20% of phospholipids are plasmalogens in human tissue, where they are particularly rich in the brain, heart, and immune cells (Lessig and Fuchs 2009; Braverman and Moser 2012). In brain, ethanolamine plasmalogens (PlsEtns) constitute approximately 60 and 80% of the total ethanolamine phospholipids in gray and white matter, respectively (Macala et al., 1983). Pls are also concentrated in specialized membranes, such as sarcolemma, myelin, and synaptic vesicles (Post et al., 1988; Takamori et al., 2006; Poitelon et al., 2020). Reduced levels of PlsEtns have been found to be associated with aging (Pradas et al., 2019) and several neurodegenerative diseases, including Alzheimer’s disease (AD) (Guan et al., 1999; Han et al., 2001; Goodenowe et al., 2007; Wood 2010; Wood et al., 2015; Yamashita et al., 2016b), Parkinson’s disease (PD) (Dragonas et al., 2009; Fabelo et al., 2011; Xicoy et al., 2019), Niemann-Pick type C disease (Schedin and Pavel 1997), multiple sclerosis (MS) (Ferreira et al., 2021) and Zellweger syndrome (Heymans et al., 1983).

**FIGURE 1:**
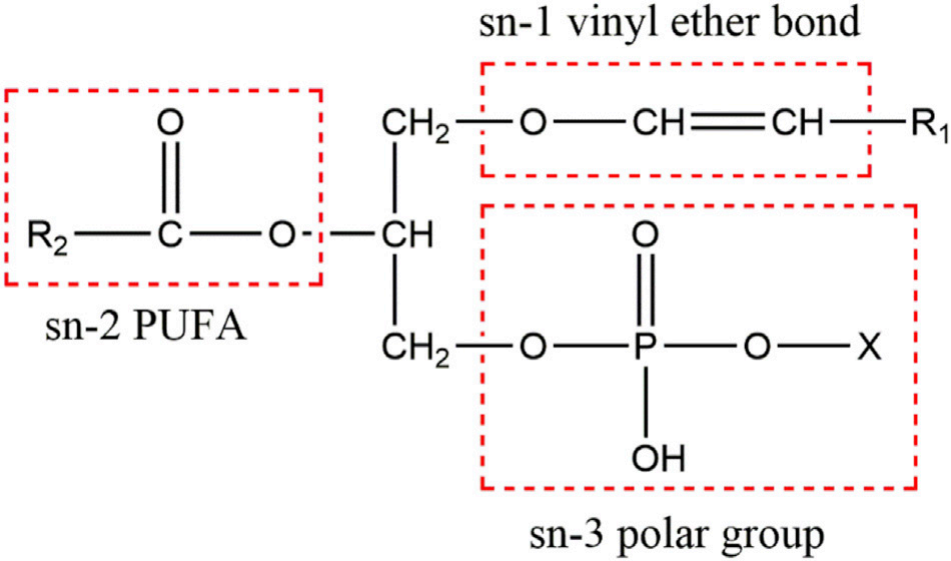
The chemical structure of plasmalogens. Plasmalogen is characterized by a vinyl ether linkage with 16:0, 18:0, and 18:1 hydrocarbon chains at sn-1 position, an ester bond linking PUFAs at sn-2 position, and a phosphate group at sn-3 position (X denotes ethanolamine or choline) of the glycerol backbone.

Plasmalogens are one of the key determinants in membrane dynamics and trafficking (Glaser and Gross 1994; Rog and Koivuniemi 2016; Skotland et al., 2019; Jimenez-Rojo and Riezman 2019; Dorninger et al., 2019). A recent structural study revealed that Pls could strongly influence the membrane thickness and curvature (Angelova et al., 2021). Due to their abundance in brain and their “fusogenic” property, plasmalogens have been proposed to play an important role in neurotransmission (Dorninger et al., 2017). The Pls-containing biomembranes have an increased propensity to undergo the transition from lamellar to non-lamellar structures, which may result in an increased leakage of membranes to ions and induction of membrane fusion (Glaser and Gross 1994; Lohner, 1996; Siegel and Epand 1997; Nagan and Zoeller 2001; Koivuniemi 2017). Plasmalogens as a major lipid component in the membranes of synapses and synaptic vesicles (Hofteig et al., 1985; Takamori et al., 2006), together with their proposed role in membrane fusion and fission processes have led to the speculations about their roles in synaptic vesicle cycle and neurotransmission (Brites et al., 2004; Han 2005; Gorgas et al., 2006; Dorninger et al., 2017). The reduction of PlsEtns may change the biophysical properties of phospholipid-bilayered cell membranes and correlate with the impairments of synaptic transmission and neurotransmitter release (Brodde et al., 2012; Dorninger et al., 2019). A neurolipidomics study showed that the aberrant PlsEtns might be involved in synaptic dysfunction in AD (Bennett et al., 2013). Moreover, plasmalogens tend to carry polyunsaturated fatty acids (PUFAs), specifically docosahexaenoic acid (DHA) or arachidonic acid (AA) at sn-2 position of glycerol backbone (Figure 1). These PUFAs may regulate the SNARE proteins, which mediate synaptic vesicle exocytosis and membrane fusion (Darios and Davletov 2006; Davletov et al., 2007; Davletov and Montecucco 2010; Bazinet and Laye 2014; Lauwers et al., 2016).

Normal function of a brain requires the formation of complex neuronal networks built based on numerous synaptic connections appropriately formed and maintained. Synaptic loss correlates strongly with cognitive decline mainly because the integrity of structure and function of synapses underlie the cognitive performance. It is widely recognized that alterations of synaptic function are not only a core feature, but also a leading cause of several neurodegenerative diseases, including AD (Selkoe 2002). The synaptic loss, triggered by amyloidosis, tauopathy and inflammation, has been considered as a potential disease biomarker in AD (Colom-Cadena et al., 2020). A recent report showed a widespread synaptic loss across the brains of early AD patients and the synapse loss was even more extensive than the decrease of volume of gray matter, suggesting the synaptic degeneration may precede the occurrence of neurodegeneration and brain atrophy (Mecca et al., 2020). Aging is thought to drive a progressive decline in synaptic connectivity of the brain, resulting in cognitive impairments and predisposition to neurodegenerative disorders (Burke and Barnes 2006; Bishop et al., 2010; Fan et al., 2017). The genetic profiling studies of aging mouse, monkey and human brains have revealed significant alterations in the expression of synapse-associated genes (Jiang et al., 2001; Lu et al., 2004; Fraser et al., 2005).

Accumulated evidence and data support that plasmalogens are able to halt neuroinflammation mediated by microglial activation both *in vitro* and *in vivo* (Ifuku et al., 2012; Hossain et al., 2018; Sejimo et al., 2018; Ali et al., 2019; Youssef et al., 2019; Nguma et al., 2021). Various inflammatory stimuli may reduce Pls levels in murine microglia, and the reduction of Pls in the murine cortex further increases the activated phenotype of microglia and also the expression of pro-inflammatory cytokines (Hossain et al., 2017). PlsEtns are also important in the phagocytosis of macrophages (Rubio et al., 2018). Microglia of aged rodent brain are less active in phagocytosis compared to those of young rodent brain (Njie et al., 2012), suggesting the reduction of brain plasmalogen level may contribute to the decline in microglial phagocytosis. The impacts of aging on both microglial and synaptic dysfunctions might be related to the reduction of plasmalogen level in the brain. Aging brains are often accompanying with the increased neuroinflammation and synaptic loss, which may attribute to aging-dependent microglial dysfunction (Niraula et al., 2017; Angelova and Brown 2019). Based on these, we thus boldly speculate that plasmalogens may act to modulate synaptic and microglial function separately in addition to microglia-synapse interaction pathways.

Thus, we investigated the effects of an ascidian-derived plasmalogens supplementation on the prevention of age-related cognitive decline in mice. In the present study, we for the first time showed that the administration of Pls could promote synaptic plasticity and neurogenesis, and inhibit the age-related microglia-mediated neuroinflammation in a natural aging mouse model. Our TEM data, immunoblotting and immunofluorescence analyses demonstrate that plasmalogens, the key brain phospholipids, improved cognition and memory by reducing neuroinflammation and supporting synaptogenesis and neurogenesis in aged mice. Our studies strongly suggest that Pls administration may serve as a potential intervention strategy for halting neurodegeneration and promoting neuroregeneration.

## 2 Materials and Methods

### 2.1 Preparation of Plasmalogens

Plasmalogens (Pls) were extracted from ascidian (*Halocynthia roretzi*) following the protocol described before (Mawatari et al., 2009). The extracted plasmalogens were analyzed and confirmed by HPLC and LC-MS methods (Mawatari et al., 2007; Otoki et al., 2017). These purified Pls are enriched with eicosapentaenoic acid (EPA), docosahexaenoic acid (DHA), and other omega-3 fatty acids. Pls were dissolved in the sterilized water to the final concentration of 50 mg/ml by sonication.

### 2.2 Animals and Study Design

7-month old (*n* = 40) female C57BL/6J mice were purchased from GemPharmatech Co. Ltd., and raised to 16-month old in Laboratory Animal Center of Shanghai Jiao Tong University. 16-month old mice were then randomly divided into two groups: aged control group and aged Pls-fed group. To discern cognitive function status in aged mice, 4-week old (n = 15) female C57BL/6J mice (GemPharmatech) were used as young control group, and raised in the same condition as aged mice. Mice in aged Pls-fed group were supplemented with Pls in water by intragastric administration for 2 months and mice in aged control group were given the same amount of water. Pls were administrated at a dosage of 300 mg/kg, once a day, and 5 days per week. Behavioral tests were performed after 2 months of the Pls treatment. At the time of behavioral tests, mice in aged Pls-fed group and aged control group were 18 months old, and mice in young controls group were 3 months old. The animal protocol was reviewed and approved by the Shanghai Jiao Tong University Institutional Animal Care and Use Committee (SJTU-IACUC). All mice were housed in a pathogen-free facility with 12-h light/12-h dark cycles, received food and water ad libitum.

### 2.3 Morris Water Maze Test

The Morris water maze test was performed as described previously (Vorhees and Williams 2006). The apparatus was a circular pool (120 cm diameter) filled with water. Tests were performed at 22°C. A 10 cm diameter transparent platform was placed 1 cm below the water surface at a fixed position. Mice were taken to the behavior room, acclimatized, and trained on five consecutive days, four trials per day. The starting point changed after each trial of a daily training session. Each trial lasted 60 s or until the mouse found the platform. If the platform was not located during the time period, then the mouse was guided to the platform, and allowed to stay there for 15 s. On day six, the platform was removed for the probe trial. The duration of probe trial was 60 s. All parameters were recorded by a video tracking system.

### 2.4 Transmission Electron Microscope Study

Mice were anesthetized with 1% pentobarbital sodium. Mice were perfused transcardially with phosphate buffered saline (PBS) followed by 4% paraformaldehyde (PFA) solution. The brains were rapidly removed and placed on ice. CA1 parts of hippocampus were dissected out immediately. Tissue blocks were first fixed in 2.5% glutaraldehyde overnight, followed by 2nd fixation of 1% osmium tetroxide for 20 min. Samples were dehydrated through an ethanol gradient and finally infiltrated and embedded in low viscosity epoxy resin kit (Spurr) containing initiator and polymerized at 70°C for 12 h. Ultrathin (50–75 nm) sections were cut with a diamond knife (DiATOME, ultra 45°) on ultramicrotome (Leica, EM UC7) and collected on 200-mesh copper grids. Sections were then stained with lead citrate for 10 min. Sections were then viewed on a transmission electron microscope (FEI, Talos F200S) at 200 kV.

### 2.5 Western Blot Analysis

Mice hippocampus were dissected after sacrificed, snap frozen and lysed in RIPA buffer (YoBiBio, China) with 1% phenylmethanesulfonyl fluoride (PMSF) (YoBiBio, China). The lysates were firstly separated on 8–12% SDS-PAGE and transferred to Polyvinylidene difluoride (PVDF) membrane. The separated proteins were immunoblotted with the following antibodies: β-Tubulin (ab6046, Abcam), Synaptophysin (ab52636, Abcam). Western blotting images were obtained by ECL exposure and quantified by Image J software. The amount of protein was expressed as a relative value to the levels of β-Tubulin.

### 2.6 RNA Sequencing Analysis

RNA purification, reverse transcription, library construction, and sequencing were performed at Shanghai Biochip Co., Ltd. (Shanghai, China) according to the manufacturer’s instructions. The expression level of each transcript was calculated according to the fragments per kilobase of exon per million mapped reads method. RNA-Seq by expectation−maximization was used to quantify gene abundances. Gene ontology (GO) annotation enrichment analysis of differentially expressed genes was analyzed at database for annotation, visualization and integrated discovery (DAVID) version 6.8 (https://david.ncifcrf.gov/home.jsp).

### 2.7 Real-Time PCR Analysis

Total RNA was extracted from the mouse brain tissue using Trizol reagent (Invitrogen), according to the manufacturer’s instructions. Reverse transcription of RNA was performed with the ReverTra Ace qPCR RT Master Mix with gDNA Remover Kit (Toyobo). The cDNA was subsequently subjected to Real-Time PCR by using SYBR Green Real-time PCR Master Mix (Toyobo). The qPCR primers used for amplifying each mouse gene were as follows: Sox2, 5′-forward CCC​ACC​TAC​AGC​ATG​TCC​TAC-3′ and reverse 5′-GCC​TCG​GAC​TTG​ACC​ACA​G-3’; IL-1β, forward 5′-GAA​ATG​CCA​CCT​TTT​GAC​AGT​G-3′ and reverse 5′-TGG​ATG​CTC​TCA​TCA​GGA​CAG-3’; TNF-α, forward 5′-AAG​CCT​GTA​GCC​CAC​GTC​GT-3′ and reverse 5′-AGG​TAC​AAC​CCA​TCG​GCT​GG-3’; IL-6, forward 5′-TAG​TCC​TTC​CTA​CCC​CAA​TTT​CC-3′ and reverse 5′-TTG​GTC​CTT​AGC​CAC​TCC​TTC-3’; GAPDH, forward 5′-CAA​TGT​GTC​CGT​CGT​GGA​TCT-3′ and reverse 5′-GTC​CTC​AGT​GTA​GCC​CAA​GAT​G-3’. The quantitative fold changes in mRNA in each sample were normalized to GAPDH expression and calculated using the 2 ^(-△△Ct)^ method.

### 2.8 Immunofluorescence Study

Anesthetized mice were perfused with PBS buffer and then with 4% paraformaldehyde (PFA) in PBS buffer. The brains were then collected in 4% PFA solution, fixed overnight at 4°C followed by the treatment with the sucrose solutions 30% until they sink. The brains were then embedded in optimum cutting temperature (O.C.T.) compound, and sectioned into 10 μm slices on a freezing microtome. Sections were incubated overnight at 4 C with primary antibodies in 3% bovine serum albumin (BSA) in PBS containing 0.1% Tween 20. Primary antibodies used in this study were rabbit anti-Synaptophysin (ab52636, Abcam), rabbit anti-Sox2 (ab93689, Abcam), rabbit anti-Iba1 (17198S, Cell Signaling Technology). Following the overnight primary antibody incubation, sections were washed five times with PBS or TBS buffer solution, incubated with the appropriate secondary antibodies (1:100, Jackson ImmunoResearch Laboratories). For quantification of the expression of Sox2, Sox2-positive cells were counted in the DG of hippocampus. For quantification of the expression of Synaptophysin, the immunofluorescence intensity was quantified using the Image J.

### 2.9 Morphometric Analysis of Iba1+ Microglia

Microglia were classified as activated when cells presented shortening of processes and increase in cell body size (dystrophic morphology) (Streit et al., 2004). The overall morphologies of Iba1+ microglia from young control, aged control and aged Pls-fed groups were analyzed with ImageJ (NIH) by using skeleton analysis and sholl analysis as described previously (Young and Morrison 2018). Iba1+ microglia were chosen randomly in the CA1 region of hippocampus, and investigators who performed tracings were blinded to the experimental groups. Simple neurite tracer plugin was used for tracing processes of microglia cells. Cells were skeletonized using 3D skeletonize plugin and labeled for slab, junctions, or end of branches. Conformational Sholl analysis was performed through concentric envelopes computed with a 1 μm radius step starting from the cell body. In each group, 30 microglia (7-8 cells/animal, 4 animals/group) were analyzed by Sholl analysis.

### 2.10 Statistical Analysis

GraphPad Prism 7.0 was used for statistical analysis. The data are shown as the mean ± SEM. Group differences were analyzed with one-way ANOVA followed by the Tukey multiple comparisons test for comparison among multiple groups. The two-tailed unpaired *t* test was applied for comparisons between two groups. Differences were considered statistically significant with *p < 0.05, **p < 0.01 and ***p < 0.001.

## 3 Results

### 3.1 Plasmalogens-Fed Intervention Improves Memory and Cognition of Aged Mice

**FIGURE 2:**
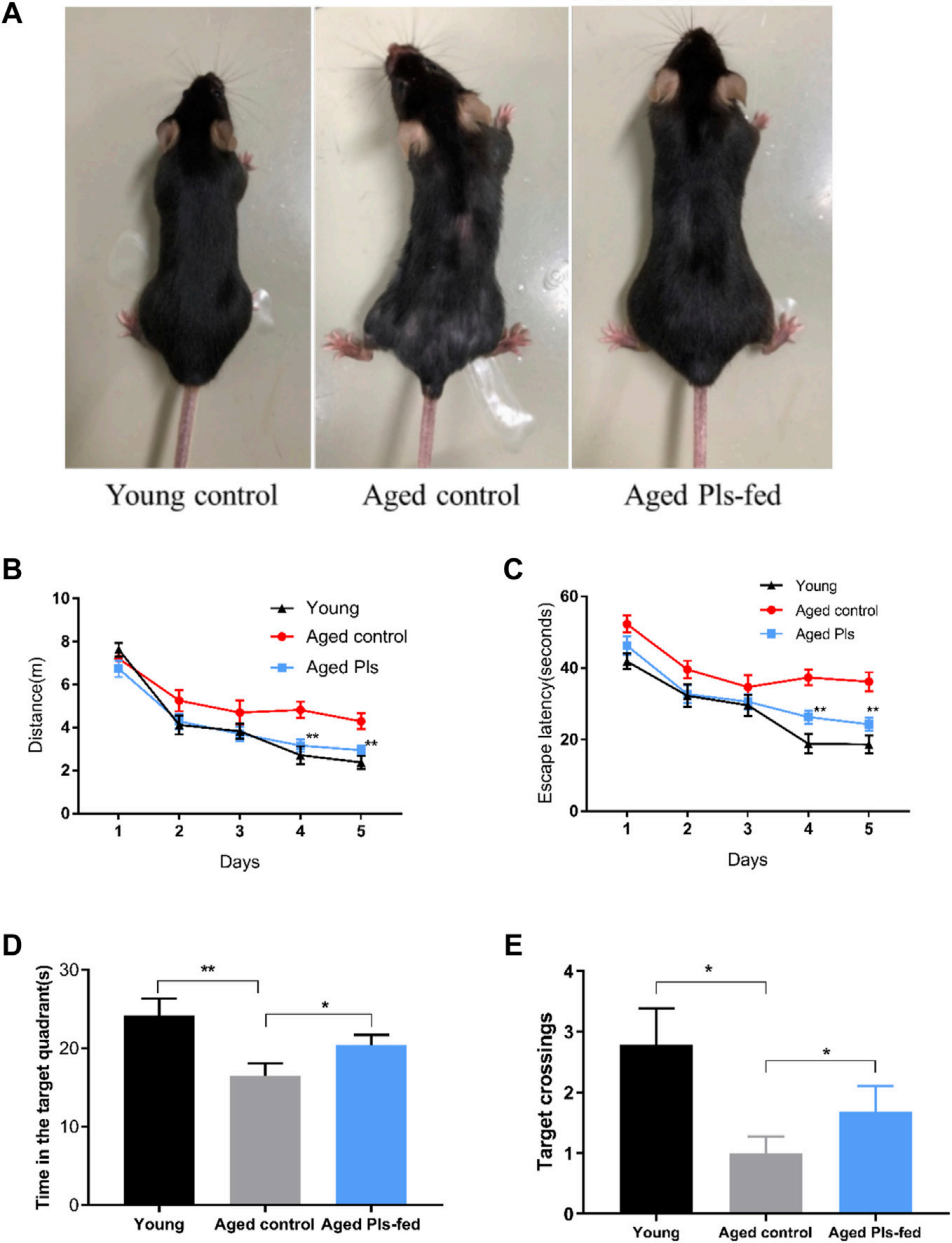
Effects of 2-months Pls supplementation on the appearance and cognitive behaviors of aged mice. **(A)** Representative photos of young mice (3 months), aged Pls-fed mice (18 months), aged control mice (18 months). **(B–E)** Spatial reference learning and memory were determined and characterized in a Morris water maze study. Mice were trained in Morris water maze for consecutive 5 days, four trials per day. The probe trials were tested on the 6th day. The distance traveled **(B)** and time spent **(C)** to reach the escape platform are demonstrated respectively. A bar graph summarizes the time spent within the target quadrant **(D)** and platform crossing numbers **(E)**. Data are presented as mean ± SEM, with 13–16 mice in each group (Young, *n* = 14; Aged control, *n* = 13; Aged Pls-fed, *n* = 16). Asterisks indicate statistical significances compared to Aged control using one-way ANOVA; Statistical significance (*p < 0.05; **p < 0.01).

In this report, we used a natural aged mice model to investigate the effect of plasmalogens (Pls) supplementation on the cognition performance. Supplementation with water or Pls by oral gavage in the aged mice started at age of 16 months and continued until age of 18 months. The body weight (Supplementary Figure S1) was not statistically different between the aged control and aged Pls-fed mice during the gavage administration period. The effect of Pls supplementation on murine cognition behavior was assessed by Morris Water Maze test. The aged mice swam a longer distance (Figure 2B) and took a longer time (Figure 2C) to find the escape platform compared to the young mice, suggesting the cognitive impairments might occur in aged mice. In comparison with aged controls, the data of aged Pls-fed mice showed a significant shorter swimming distance and escape latency to reach the escape platform on the 4th day and 5th day (Figures 2B,C). In the probe trial, with the platform removed, trials were performed to evaluate the spatial reference memory of the mice. The aged Pls-fed mice spent more time in the target quadrant and the crossing numbers were significantly increased over that of aged control mice (Figures 2D,E). These outcomes reveal that aged mice with 2-months Pls supplementation have better spatial learning and memory capacity compared to aged control mice. Of note, by the age of 18 months, the appearance of aged control mice showed striking signs of senescence, characterized by gray body hair and obvious hair loss, while aged Pls-fed mice look healthy in appearance with glossier and thicker hair compared to aged controls (Figure 2A). These data suggest that Pls supplementation may alleviate age-related cognitive decline and reverse the aged symptoms in mice.

### 3.2 Plasmalogen Treatment Alleviates Age-dependent Synaptic Loss in Murine Hippocampus

**FIGURE 3:**
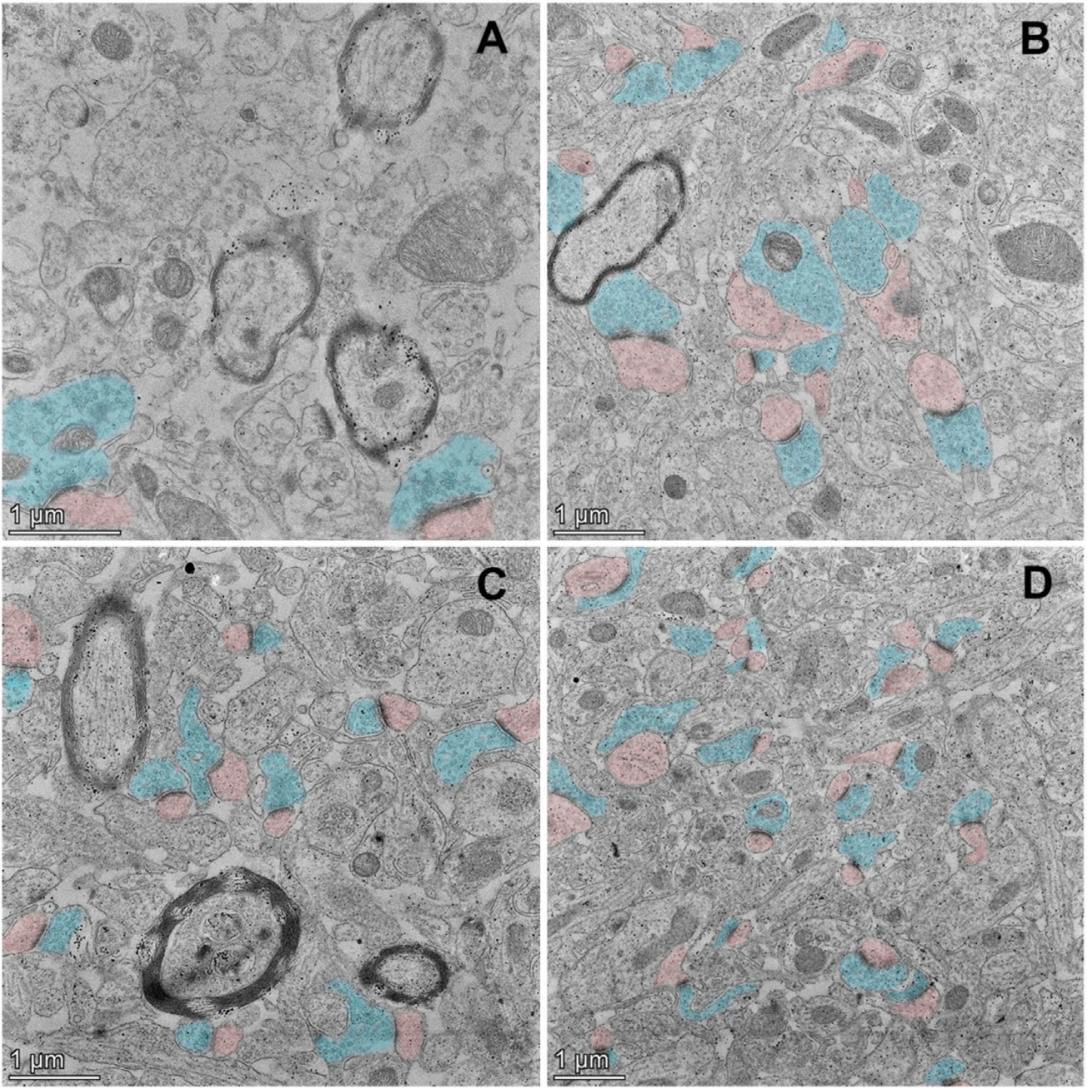
TEM ultrastructural study on murine hippocampal CA1 region. The TEM micrographs of aged control mice **(A)**, young control mice **(B)**, and aged Pls-fed mice **(C,D)**. Pre-synaptic terminals are marked with blue color, and post-synaptic domains with pink color. The synaptic loss, synaptic degradation and deterioration were observed in aged controls **(A)**. On the contrary, abundant synaptic vesicles are present in the pre-synaptic terminals (marked in blue) of young controls **(B)**. There is a pronounced increase in the numbers and density of synapses in aged Pls-fed murine hippocampus with moderate numbers of synaptic vesicles **(C,D)**. Bars = 1 μm.

Synapses are specialized intercellular junctions with two apposed compartments, pre-synaptic terminal and post-synaptic domain, and synaptic cleft, a gap about 20 nm between pre- and post-synaptic components (Hernandez-Nicaise 1973). As shown in Figure 3, pre-synaptic domains are marked with blue color, and post-synaptic domains with pink color. The typical ultrastructures of synapse of young control (Figure 3B) and aged Pls-fed mice (Figures 3C,D) were examined by TEM. Remarkably, abundant synaptic structures are present in both young controls (Figure 3B) and aged Pls-fed mice (Figures 3C,D), and the numbers of synapse are far more than we have observed in those of aged controls (Figure 3A). There are plenty of synaptic vesicles in the presynaptic domains in both young controls and aged Pls-fed mice. Although young controls (Figure 3B) and aged Pls-fed mice (Figures 3C,D) have similar and comparable numbers of synapses, the number of synaptic vesicles in young mice is greater compared to aged Pls-fed group in the total viewed TEM images (each group 30–40 images from 3-4 biologically independent samples), nevertheless both young controls (Figure 3B) and aged Pls-fed mice (Figures 3C,D) have much greater numbers of synaptic vesicles compared to aged controls (Figure 3A). In aged control samples, synaptic loss is visually obvious and synaptic ultrastructures showed severe deterioration with few synaptic vesicles (Figure 3A). In aged Pls-fed mice (Figures 3C,D), the synaptic structures are intact and look similar to those of young controls (Figure 3B), with synaptic vesicles evenly distributed in the presynaptic domains. The TEM data strongly indicate that Pls supplementation may protect against the hippocampal synaptic loss in aged mice.

### 3.3 Plasmalogens Treatment Changed the Transcriptomics Profile of Hippocampus in Aging Mice

**FIGURE 4:**
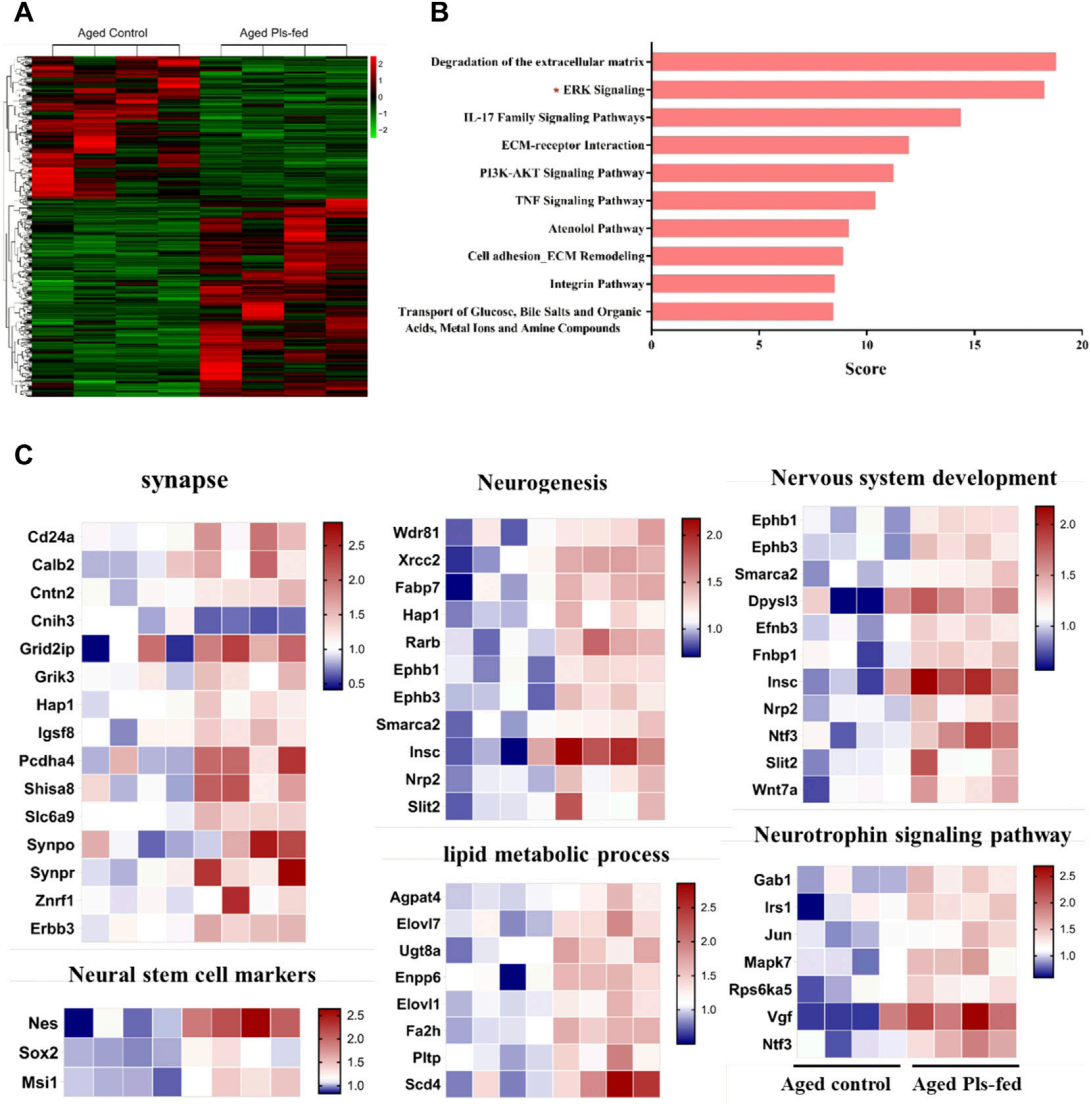
Transcriptomics profile of hippocampus in aged Pls-fed mice. **(A)** A heat map shows all differentially expressed genes in aged control versus aged Pls-fed murine hippocampus based on RNA-seq (*n* = 4 mice per group). **(B)** GeneAnalytics pathway analysis of differentially expressed genes in hippocampus of aged control vs. aged Pls-fed mice, the top ten pathways are highlighted here. **(C)** Plasmalogen treatment changed the transcriptomics profile of hippocampus in the aged mice. (*n* = 4 mice per group).

To investigate the molecular mechanism of plasmalogens improving learning and memory of aged mice, transcriptome analysis was performed by RNA-sequencing (RNA-seq) on the experimental murine hippocampus tissues. There are 802 differentially expressed genes (Ratio >1.2 or Ratio <0.8, and *p* < 0.05) in the aged Pls-fed mice compared with aged control (Supplementary Table S1), and heatmap analysis of all the differentially expressed genes was shown in Figure 4A. GeneAnalytics pathway analysis showed that these differentially expressed genes were involved in several pathways, including ERK, PI3K-AKT signaling (Figure 4B). The DAVID version 6.8 was used for function annotation analysis and enrichment analysis, which shown that these genes were engaged in synapse (Synpo, Synpr, Erbb3, and Slc6a9, etc.), neurogenesis (Fabp7, Nrp2, Ephb1, and Insc, etc.), neural stem cell (Nestin, Sox2, and Msi1), nervous system development (Ephb3, Dpysl3, Smarca2, and Efnb3, etc.), neuotrophin signaling pathway (Vgf, Ntf3, Gab1, and Irs1, etc.), lipid metabolic process (Agpat4, Elovl1, Elovl7, and Fa2h, etc.) (Figure 4C). Neurotrophins including VGF and neurotrophin-3 (Ntf-3), are considered powerful molecular mediators in synaptic plasticity (Poo 2001; Park and Poo 2013). The elevated level of neurotrophins in aged Pls-fed mice may contribute to potential neurogenesis and observed synaptogenesis in aged Pls-fed murine hippocampus. These RNA-sequencing data point out that Pls administration may upregulate the expression of synapse-associated genes and promote synaptogenesis and neurogenesis in aged murine hippocampus.

### 3.4 Plasmalogens Enhance the Hippocampal Synaptic Plasticity in the Aged Mice

**FIGURE 5:**
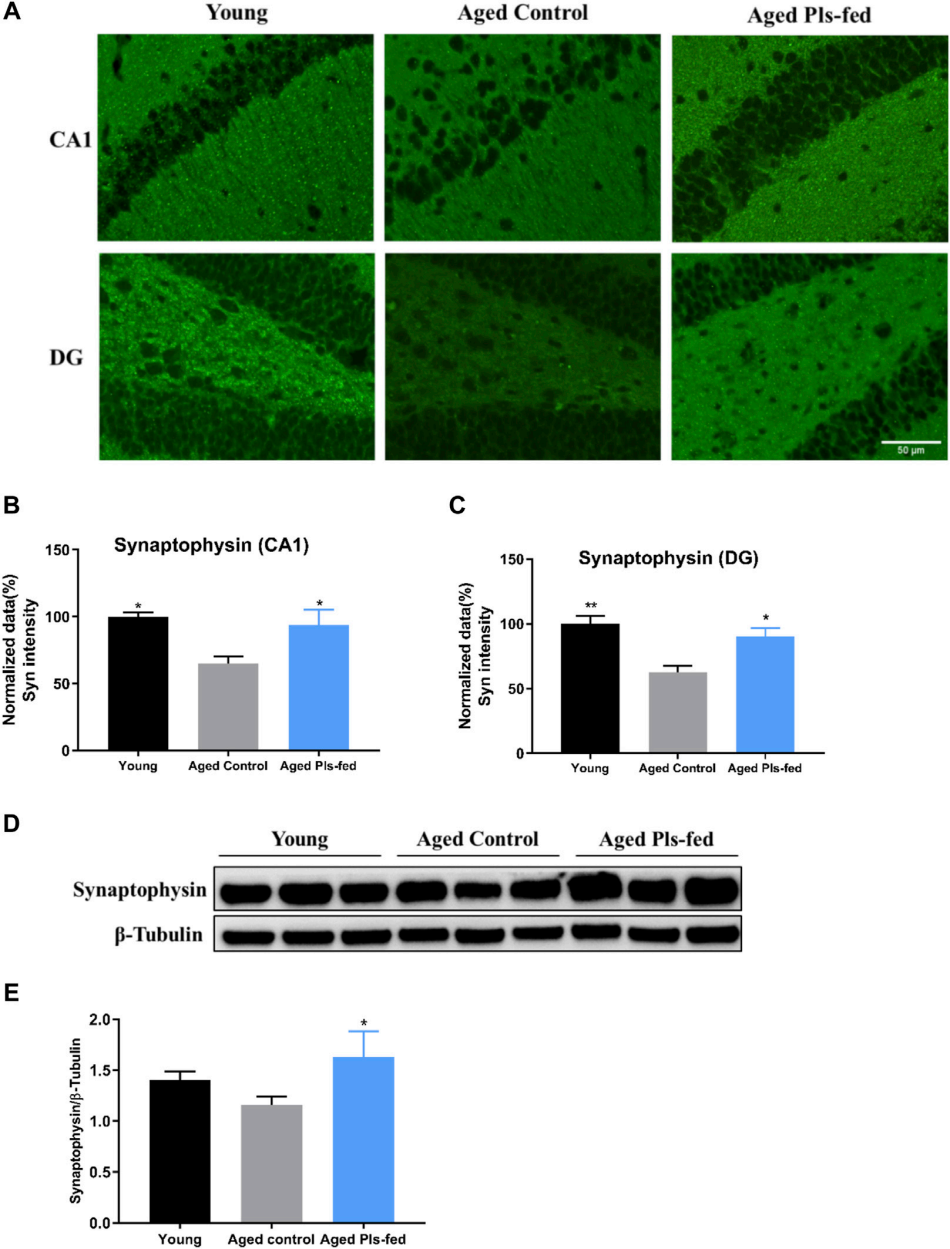
Plasmalogens enhance synaptic plasticity in the hippocampus of aged mice. **(A)** Representative images showing the expression of synaptophysin (green) in the CA1 and DG region of mice hippocampus. **(B–C)** Graph showing quantification of the immunostaining of synaptophysin in the CA1 and DG region of murine hippocampus (*n* = 4 mice for each group), and values are presented as mean ± SEM. **(D)** Representative immunoblotting of synaptophysin levels and **(E)** graph showing quantification of synaptophysin levels. The quantification is a ratio of synaptophysin/β-tubulin levels (*n* = 3 mice per group), and values are presented as mean ± SD. Asterisks indicate statistical significances compared to Aged control by one-way ANOVA; significance (*p < 0.05; **p < 0.01), scale bars, 50 μm.

Learning and memory are associated with the remodeling and growth of synapses (Bailey et al., 2015). Above-mentioned TEM and RNA-seq results have suggested that Pls may enhance the hippocampal synaptic plasticity. To further verify the alterations of synaptic plasticity, we investigated the expression of synaptic plasticity-related protein, synaptophysin, by immunoblotting and immunofluorescence techniques. Immunofluorescence studies showed that, the expression of synaptophysin was significantly reduced in the CA1 and DG regions of hippocampus of aged controls mice compared with that of young mice, and significantly increased in aged Pls-fed mice compared with that of the aged controls (Figures 5A–C). Furthermore, immunoblotting analysis also showed that synaptophysin was markedly upregulated in the hippocampus of aged Pls-fed mice (Figures 5D,E). These results indicate that synaptic plasticity decreases in an age-related manner and can be enhanced by Pls supplementation.

### 3.5 Plasmalogens Promote Neurogenesis in the Aged Murine Hippocampus

**FIGURE 6:**
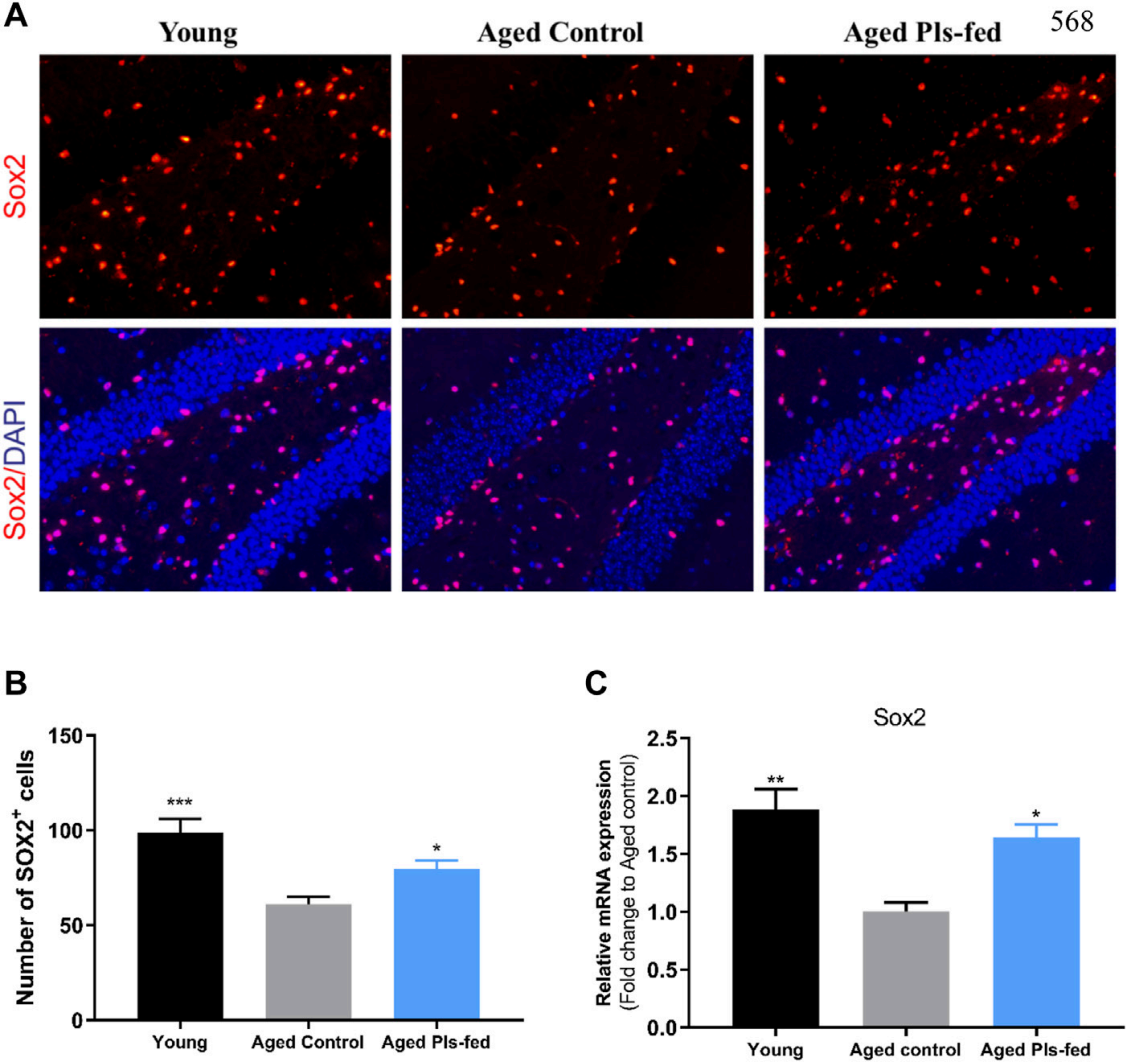
Plasmalogens promote neurogenesis in the aged murine hippocampus. **(A)** Representative fluorescence images showing neural stem cells in young control, aged control and aged Pls-fed mice of hippocampal dentate gyrus (DG). Cells are identified based on anti-Sox2 (red). DAPI indicates nuclear staining (blue). **(B)** graph showed quantification of Sox2+ cells among groups. **(C)** The real-time PCR data showed mRNA expression of Sox2 in hippocampus of young control, aged control and aged Pls-fed mice. All values are presented as mean ± SEM. Asterisks indicate statistical significances compared to Aged control by one-way ANOVA; significance (*p < 0.05; **p < 0.01; ***p < 0.001), scale bars, 50 μm n = 4 mice for each group.

In the aged brain, neurogenesis decline leading to reduced neuroplasticity and cognitive function (Villeda et al., 2011). Adult neurogenesis usually occurs in neurogenic niches in the dentate gyrus (DG) of hippocampus (Villeda et al., 2011). To further investigate whether plasmalogens can affect the neurogenesis, we examined the proliferative Sox2+ stem cells in the murine hippocampal sections. Immunofluorescence data showed that, the number of Sox2+ stem cells was significantly reduced in the DG regions of hippocampus of aged controls compared with that of young controls, and significantly increased in the aged Pls-fed mice compared with that of the aged controls (Figures 6A,B). Furthermore, the increased Sox2 mRNA level (Figure 6C) in the aged Pls-fed mice compared with that of the aged controls is consistent with previous RNA-seq data (Figure 4C). These results demonstrate that plasmalogens may activate the NSCs proliferation, which is beneficial to stabilize the neural network in hippocampus.

### 3.6 Plasmalogens Suppress the Microglia Activation and Attenuate Neuroinflammation in the Aged Murine Brain

**FIGURE 7:**
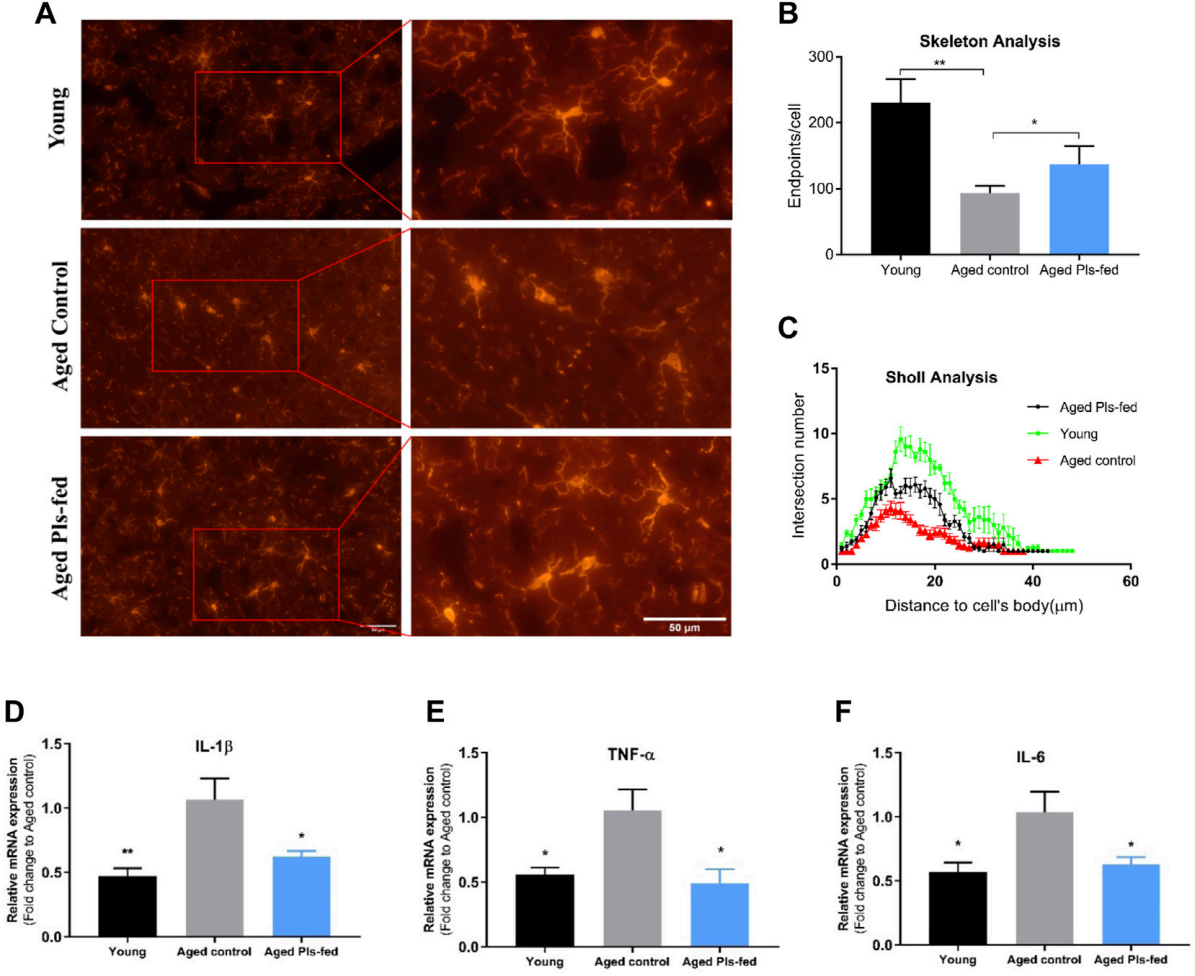
Effects of plasmalogens intervention on microglia activation and inflammatory cytokines expression. **(A)** Immunofluorescence staining of Iba1+ microglia cells (red) in the hippocampus of young control, aged control and aged Pls-fed mice, scale bars, 50 μm. Note that microglia of aged control mice display shorter processes and diminished ramification of processes, whereas microglia of aged Pls-fed and young control show longer and ramified processes. **(B)** The graph bar of skeleton analysis on microglia showed that the endpoints are increased in microglia of aged Pls-fed mice. **(C)** Sholl analysis of the number of branches plotted as a distance from cell body (*n* = 30, microglial cells per experimental group). **(D–F)** The real-time PCR data showed mRNA expression of pro-inflammatory cytokines IL-1β, TNF-α, IL-6 in hippocampus of young control, aged control and aged Pls-fed mice. All values are presented as mean ± SEM. Asterisks indicate statistical significances compared to Aged control by one-way ANOVA (*p < 0.05; **p < 0.01).

Microglia partake in many important events in adult brain including neurogenesis, synaptic plasticity, synaptic pruning and maintenance of neuronal health (Kierdorf and Prinz 2017). In healthy brain, microglia exhibit a ramified morphology, termed as “resting” or “surveying” microglia. Whereas microglia in the aged brain display a dystrophic morphology, classified as a “primed” or “sensitized” phenotype (Niraula et al., 2017; Hanisch and Kettenmann 2007). Immunofluorescence analysis of Iba1+ cells allowed us to view microglial shapes in the murine hippocampus (Figure 7A). The morphological features of surveying microglia with branched long processes were observed in young control and aged Pls-fed samples, whereas the dystrophic microglia were observed in aged control mice (Figure 7A). Quantitative analysis of morphological changes of microglia demonstrated the noticeable differences among three groups. The skeleton analysis data showed that endpoints were lower in the aged control compared to young control and aged Pls-fed mice (Figure 7B). The Sholl analysis data showed an increase in the number of branches in microglia of aged Pls-fed mice compared to those of aged controls (Figure 7C). The experimental results indicate that plasmalogens may alleviate age-related microglia activation. The primed phenotype of microglia in the aged brain may produce more pro-inflammatory cytokines such as TNF-α, IL-1β and IL-6 (Sierra et al., 2007). The measurements of pro-inflammatory cytokines, revealed that the levels of IL-1β, IL-6 and TNF-α (Figures 7D–F) were significantly reduced in the hippocampal tissues of aged Pls-fed mice compared to aged controls. These experimental outcomes demonstrate that plasmalogens may inhibit microglial activation and reduce pro-inflammatory cytokines in the aged mice.

## 4 Discussion

There has been an increasing interest in plasmalogens research due to the emerging roles they play in health and diseases (Su et al., 2019; Dorninger et al., 2020; Bozelli et al., 2021). The reduced level of plasmalogens has been commonly observed in the patients suffering from neurodegenerative disorders and appears to meet Bradford Hill criteria for causal association with neurodegeneration to a considerable extent (Senanayake and Goodenowe 2019). Moreover, plasmalogen replacement therapy (PRT) has been shown to be a successful way to restore plasmalogen levels as well as to ameliorate pathological phenotypes (Bozelli and Epand 2021), and an increasing number of studies suggests that plasmalogens may serve as potential therapeutic intervention for multiple neurodegenerative diseases, including AD and PD (Fujino et al., 2017; Yamashita et al., 2017; Bourque et al., 2018; Fujino et al., 2018; Nadeau et al., 2019; Mawatari et al., 2020). These reports all point to a fact that the significance of plasmalogens in neuro-degeneration and neuro-regeneration. However, these studies also raised some puzzling questions about the actual mechanisms of plasmalogens that remain to be revealed. Based on others’ and our own experimental data, we thus boldly propose that plasmalogens may improve age-related cognitive decline through the promotion of neurogenesis, especially synaptogenesis and synaptic vesicles formation to counteract the loss of synaptic connectivity and networking in aging and neurodegenerative process.

In the present study, we attempt to answer these important questions by examining the synaptic and microglial response to plasmalogens treatment in the aged female mice. The decline of cognitive function in humans is progressive, usually starting at middle age (Schönknecht et al., 2005). Compared to males, age-related cognitive decline and brain atrophy occur earlier in females (Yuan et al., 2012; Podgórski et al., 2021). Women have a higher life expectancy than men, but reported a higher prevalence of dementia (Corrada et al., 2008). For these reasons, naturally aged female mice were used as experimental subjects in this study. The 18-month old C57BL/6J mice showed significant cognitive impairments compared to young controls, while the aged Pls-fed mice have significantly better spatial learning and memory performance than that of aged controls. Moreover, aged Pls-fed mice look healthy in appearance with glossier and thicker body hair comparable to young control mice. From our close observations, the aged mice began to grow new black hair clearly visible to the naked eyes since the 3rd week of Pls supplementation. As Pls administration was continuously introduced to the aged mice, the black hair became thicker with glossy look. The intragastric administration of plasmalogens effectively improved the physiological structure and function of synapses in the aged murine hippocampus and might further reverse the age-related cognitive functions of the brains.

Aging is thought to drive a progressive decline in synaptic connectivity and neurogenesis in brain, resulting in cognitive impairments and predisposition to neurodegenerative disorders (Burke and Barnes 2006; Dickstein et al., 2007; Rossi et al., 2008; Villeda et al., 2011; Fan et al., 2017). Synaptic loss is correlated most strongly with cognitive decline and synaptic function; the latter is underlying the cognitive performance. In this report, the hippocampal synaptic ultrastructures of CA1 area in aged Pls-fed mice appear comparable to those of young controls. Whereas the number and density of synapses and synaptic vesicles of aged controls are visibly much less compared to those of young controls and aged Pls-fed mice. Our observations of synaptic damage and loss in the aged controls are consistent with that of previous reports (Bondareff and Geinisman 1976; Geinisman et al., 1986; Geinisman et al., 1992). TEM data in this report provide the first direct visual evidence that plasmalogens supplementations may alleviate age-dependent synaptic loss and also promote synaptogenesis and synaptic vesicles (SVs) formation. Therefore, we further examined neurogenesis and synaptic plasticity associated proteins. Synaptophysin is a synaptic vesicle glycoprotein, involved in the fusion of neurotransmitter vesicles and regulation of synaptic vesicle exocytosis (Südhof 1995; Janz et al., 1999). The markedly increased expression of synaptophysin in aged Pls-fed mice, suggests that plasmalogens as a major lipid component of synapses may play role in promoting the formation of SVs and synaptic membrane. Moreover, plasmalogens can activate the neural stem cells and promote neurogenesis in aged murine brain, and both may have significant effects on maintaining the cognitive function of animals. Furthermore, RNA-seq analysis on the murine hippocampus revealed the obvious changes in synapse, neurotrophins, neurogenesis, neural stem cell and lipid metabolism at the genetic and molecular level in response to plasmalogen administration. All these findings support that Pls may reverse cognitive aging by enhancing the synaptic plasticity and neurogenesis in aging mice.

During aging process, activated microglia release a plethora of cytokines, chemokines, and reactive oxygen species, which in turn affect various aspects of adult neurogenesis (Sierra et al., 2010; Harry 2013). The continuous over-activated microglia may lead to the development of neurodegeneration. Microglia in the aged brain have been characterized by the dystrophic morphologies (Niraula et al., 2017), which are arguably less capable in securing CNS homeostasis and may trigger the neuroinflammation to further develop neurodegenerative disorders. The dystrophic microglia as an indicator of pathological precursor may contribute to overall weakening of synaptic connectivity and plasticity, often underlying age-dependent cognitive decline. Downregulation of plasmalogens synthesis by injection of lentiviral shRNAs targeting the Gnpat mRNA causes activated NF-κB in the cerebral cortex of mice, an observation accompanied by a pro-inflammatory state of microglia in the manipulated brain region (Hossain et al., 2017). Vice versa, complementary experiments *in vitro* and *in vivo* indicate that plasmalogens supplementation can halt inflammatory response, through shifting microglia cell to a less pro-inflammatory state (Hossain et al., 2018; Sejimo et al., 2018; Ali et al., 2019; Youssef et al., 2019). In our study, microglia in aged Pls-fed mice brain are less activated (Figures 7A,B), suggesting that plasmalogens may alleviate age-related microglial activation and retain them in a surveillance state.

Neuroinflammation is a common feature of multiple neurodegenerative disorders, including AD. Neuroinflammation is known as a negative regulator of adult hippocampal neurogenesis (Ekdahl et al., 2003; Monje et al., 2003) and its progressive enhancement in hippocampus has been considered as one of the hallmarks of aging. The aged microglia produce more pro-inflammatory cytokines including TNF-α, IL1-β, and IL-6 (Sierra et al., 2007). Our examinations on microglia-mediated pro-inflammatory cytokines (IL-1β, IL-6 and TNF-α), demonstrated a significant decline of these inflammatory factors in the hippocampus of aged Pls-fed mice compared to that of aged controls. The reduced neuroinflammation mediated by plasmalogens supplementation in aged brain may help maintain a healthy nervous system and against cognitive decline.

The bioavailability of oral supplements of plasmalogens to the neuronal cells is still not clear. Although some studies suggest that plasmalogen precursors can pass through the blood-brain barrier (BBB) (Wood et al., 2011; Malheiro et al., 2019) the penetration of plasmalogens across BBB may be low in efficacy (Fallatah et al., 2020). Plasmalogen can be hydrolysed into PUFA and lyso-plasmalogen by gut phospholipase A2 (Jurkowitz et al., 1999; Wu et al., 2011). Lyso-plasmalogen could be transported to various tissues and organs and be reused to synthesize new plasmalogen species (Yamashita et al., 2021). Plasmalogens from ascidians (*Halocynthia roretzi*) contain unsaturated fatty acids such as DHA and EPA (Yamashita et al., 2014; Yamashita et al., 2016a). One study has suggested that the phospholipid form of DHA could cross the blood-brain barrier at a rate of approximately 10 times faster than that of the free fatty acid form of DHA (Lagarde et al., 2015). Therefore, the ingestion of plasmalogens with DHA might exert enhanced neuroprotective and neural function maintenance effects in the brain. Further studies are necessary to examine whether 2 months of oral Pls supplements may improve the plasmalogen levels and/or change the plasmalogen profiles or fatty acids profiles in the aged brain.

In conclusion, our results in the aging mouse model provide novel evidence that ascidian-derived plasmalogens treatment for 2 months is effective in improving cognitive function of the elderly. Further analyses of suggested that improved cognitive function following Pls treatment is linked to the enhanced synaptic plasticity, anti-inflammatory effects, and enhanced neurogenesis in the aged hippocampus. These results suggest that administration of plasmalogens may offer a promising strategy to improve cognitive function as we age.

## Data Availability

The datasets presented in this study can be found in online repositories. The names of the repository/repositories and accession number(s) can be found below: https://www.ncbi.nlm.nih.gov/bioproject/PRJNA781063.
