# Supplementary figures and images for: Plasmalogens Eliminate Aging-Associated Synaptic Defects and Microglia-Mediated Neuroinflammation in Mice
Source: Front Mol Biosci. 2022 Feb 23;9:815320. doi: 10.3389/fmolb.2022.815320 (PMC8906368; doi:10.3389/fmolb.2022.815320)

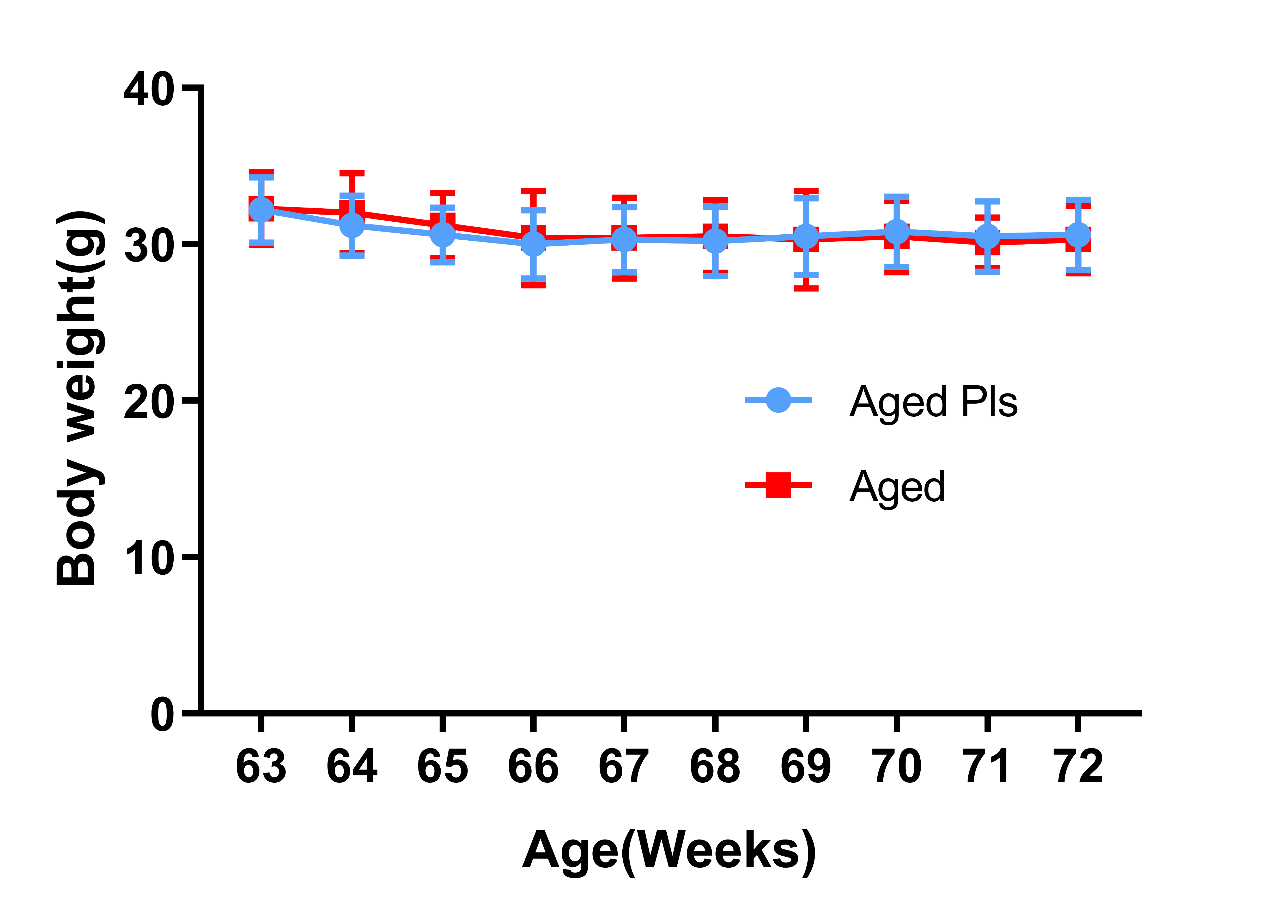

Supplement: Supplementary file 1 [file Image1.TIF]
